# Bimodal Microstructure and Strengthening Mechanisms of a Mg-RE Alloy Processed by Asymmetric Upsetting–Extrusion

**DOI:** 10.3390/ma18215001

**Published:** 2025-11-01

**Authors:** Nanjiang Chen, Bingchun Jiang, Yuze Xi, Lei Jing, Liwei Lu, Yiquan Li

**Affiliations:** 1 School of Mechanical and Electrical Engineering, Guangdong University of Science and Technology, Dongguan 523083, China; cnj0707@163.com; 2 Sanya Institute of Hunan University of Science and Technology, Sanya 572024, China; 13273372811@163.com (Y.X.); cqullw@163.com (L.L.); 3 Northwest Nonferrous Metal Baoji Innovation Research Institute, Baoji 721000, China; zfjinglei@163.com; 4 School of Materials Science and Engineering, Hunan University of Science and Technology, Xiangtan 411201, China

**Keywords:** magnesium alloy, upsetting–extrusion, bimodal microstructure, fiber texture, high strength

## Abstract

This study successfully produced a magnesium alloy bar featuring a bimodal microstructure with high strength via an asymmetric upsetting–extrusion process. The evolution of microstructure, texture, and mechanical properties was systematically investigated using finite element simulation, room-temperature tensile tests, optical microscopy, scanning electron microscopy, and electron backscatter diffraction. Results demonstrate that the bimodal structure forms under the combined effects of shear deformation in the upsetting stage and low-speed, high-ratio deformation in the extrusion stage. This structure consists of coarse deformed grains containing high-density dislocations surrounded by fine dynamically recrystallized grains. A strong <10-10>//ED basal fiber texture also developed, which effectively suppresses basal slip. Continuous dynamic recrystallization was the primary grain refinement mechanism. The 370 °C extruded alloy achieved a high tensile strength of 457.9 MPa, but its elongation was limited to 3.96%. This combination of strength and ductility is attributed to the synergistic influence of the bimodal microstructure, strong basal texture, and high dislocation density.

## 1. Introduction

Magnesium alloys, as the lightest metallic structural materials, demonstrate significant potential for lightweight applications in aerospace, transportation, electronic communication, and national defense industries due to their excellent properties, including high specific strength and stiffness, good damping capacity, strong electromagnetic shielding, and recyclability [1,2]. They are regarded as one of the most promising green engineering materials in the 21st century. However, the hexagonal close-packed crystal structure of magnesium limits the number of independent slip systems available at room temperature, with plastic deformation primarily relying on basal <a> slip. This results in relatively low absolute strength, poor room-temperature ductility, and limited formability [3]. These inherent disadvantages severely restrict the widespread application of magnesium alloys as critical load-bearing structural components. To address these issues, refining grain size and controlling texture through plastic deformation have proven to be fundamental and effective strategies for improving mechanical properties. Hot extrusion is among the most widely used and critical processes for producing high-performance magnesium alloys [4].

In this context, upsetting extrusion (UE), as a prominent variant of conventional hot extrusion, has attracted considerable research attention due to its superior capability in achieving significant grain refinement and desirable texture control. Zhang et al. [5] reported that in Mg-Gd-Y-Zn-Zr alloy, a high height-to-diameter ratio UE markedly refines grain structure and strengthens texture, achieving a ultimate tensile strength (UTS) of 355 MPa with an elongation (EL) of 17%. Wu et al. [6] demonstrated that modifying the deformation path during repetitive UE of a similar material obtained a UTS of 382.5 MPa and an EL of 8.7%. Han et al. [7] observed that isothermal repetitive UE of VW93A magnesium alloy induces more complete dynamic recrystallization (DRX), leading to significantly refined and homogeneous grains.

Bimodal microstructures frequently occur in extruded magnesium alloys and represent a key microstructural feature for achieving a synergistic combination of strength and ductility. Zhang et al. [8] produced a high-strength Mg alloy with low rare-earth content via extrusion, which exhibited a bimodal microstructure consisting of fine equiaxed DRXed grains with an average size of 0.82 μm. This alloy achieved a yield strength (YS) of 408 MPa and an EL of 6.4%. In such bimodal structures, the high dislocation density and strong texture in the unDRXed regions primarily contribute to the high YS, while the DRXed grains enhance ductility by accommodating plastic deformation, thereby improving both strength and ductility [9,10,11,12]. Another typical characteristic of extruded magnesium alloys is the formation of a <10-10>//ED basal fiber texture, which leads to high strength, low ductility, and strong anisotropy. Tang et al. [13] observed a strong <10-10>//ED texture in the weld zone of an extruded ZK60 magnesium plate, which resulted in a UTS of 315 MPa along the extrusion direction (ED). Pan et al. [14] achieved a UTS of 460 MPa in an Mg-2Sn-2Ca alloy through conventional low-temperature and low-speed extrusion, and identified the presence of a <10-10>//ED texture within the unDRXed grains. The existence of the <10-10>//ED texture aligns the basal planes of the grains parallel to the ED. When tensile stress is applied along the ED, basal slip is strongly inhibited due to the low Schmid factor, necessitating the activation of non-basal slip systems with higher critical resolved shear stresses (CRSS). This mechanism significantly enhances the strength along the ED [15,16,17].

This study investigates a Mg-3.3Gd-2.5Nd-0.4Zn-0.3Zr-0.2Sm alloy processed by an asymmetric upsetting–extrusion (AUE) technique using a horizontal extrusion machine (Wanxin, Zhengzhou, China). The microstructure, texture, and mechanical properties were systematically characterized through finite element simulation, room-temperature tensile testing, optical microscopy (OM), scanning electron microscopy (SEM), and electron backscatter diffraction (EBSD). The ultra-high strength and limited ductility were elucidated based on the bimodal microstructure, strong texture, and Schmid factor analysis, while the grain refinement mechanisms were clarified. This work aims to provide new process guidelines for designing high-performance extruded magnesium alloys, reveal the underlying mechanisms, and expand the application prospects of magnesium alloys.

## 2. Experimental Procedure

### 2.1. Sample Preparation

The experimental material employed in this study was a rare-earth (RE) magnesium alloy with a nominal chemical composition of Mg-3.3Gd-2.5Nd-0.4Zn-0.3Zr-0.2Sm (wt%) (supplied by Hunan Rongtuo New Material Research Co., Ltd., Xiangtan, China). The as-cast billets were subjected to solution treatment at 540 °C for 8 h to eliminate partial casting defects and facilitate the dissolution of secondary phases into the matrix as much as possible.

### 2.2. Finite Element Simulation

The finite element simulation of the AUE process was performed using Deform-3D software (Version 11.0). The die and punch were defined as rigid bodies, while the billet was modeled as a plastic body and discretized into 80,000 tetrahedral elements. Remeshing was activated when the relative interference depth reached 0.7. Both the tooling and the billet were maintained at 370 °C, and the punch speed was set to 0.2 mm/s. The simulation consisted of 90 steps, with a step increment of 0.1 mm per step. A shear friction model with a coefficient of 0.3 was applied at the interfaces between the billet and the tooling. The simulation employed a Lagrangian incremental type, a conjugate gradient solver, and a direct iteration method.

### 2.3. AUE Process

**Figure 1:**
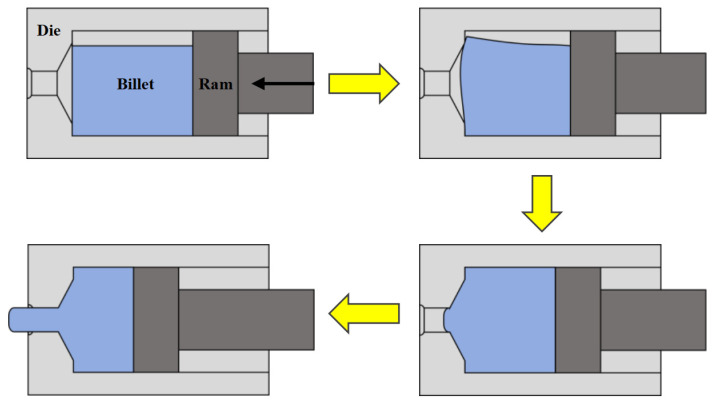
Schematic diagram of AUE die structure and experimental.

Solution-treated samples were processed using a horizontal extrusion machine. The die cavity and billets were cylindrical in geometry. The diameters of the die cavity, billet, and bar were 35 mm, 30 mm, and 8 mm, respectively, corresponding to an extrusion ratio of 19.14. Experiment was conducted at 370 °C and 410 °C with a punch speed of 0.2 mm/s. The outlet of the die remained unobstructed throughout the process. Extrusion began only after the billet had completely filled the die cavity and the reducing zone via upsetting. A schematic diagram of the AUE process is presented in Figure 1.

### 2.4. Microscopic Characterization Experiment

**Figure 2:**
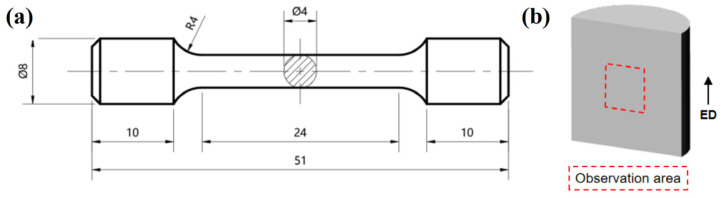
(**a**) Dimensions of tensile test specimens (mm); (**b**) Metallographic and EBSD observation areas of the extrusion specimen.

The microstructures of the solution-treated and as-extruded alloys were characterized using OM. The specimens were successively ground with 320#, 800#, 1200#, and 2000# grit sandpapers, followed by mechanical polishing with diamond polishing paste. Finally, they were etched with a solution composed of 2.5 g picric acid, 2.5 mL acetic acid, 50 mL ethanol, and 50 mL distilled water prior to OM observation. The solution-treated alloy was further examined to identify the residual secondary phases using a Tescan Mira4 field emission SEM (Tescan, Brno, Czech Republic) equipped with an Oxford Xplore 30 EDS detector (Oxford Instruments, Oxford, UK). The formed bars were machined into cylindrical tensile specimens with the geometry shown in Figure 2a. Room-temperature tensile tests were then conducted on an Instron 3369 universal testing machine (Instron, Norwood, MA, USA) at a speed of 2 mm/min. For EBSD analysis, samples were sectioned parallel to the ED from the extruded bar using wire electrical discharge machining (Shuanghua, Taizhou, China). Specimens for EBSD were taken from the central region of the axial section. The EBSD examination was performed using an Oxford Symmetry S3 detector (Oxford Instruments, Oxford, UK) operating at 10 kV, with a step size of 0.3 μm. The acquired data were processed using the Channel 5 software (Version 5.0.9.0). The specific sampling locations for metallographic and EBSD analyses are illustrated in Figure 2b.

## 3. Results and Discussion

### 3.1. Finite Element Analysis

**Figure 3:**
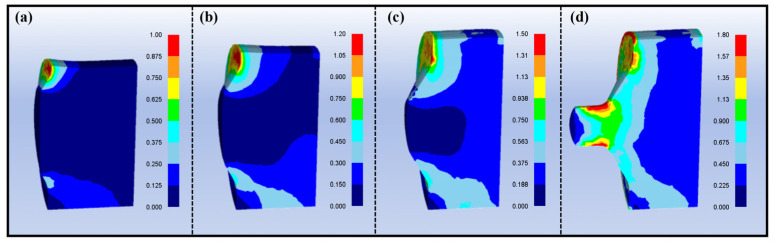
Effective strain distribution of axial-section: (**a**) Step 30, (**b**) Step 50, (**c**) Step 69, (**d**) Step 75.

Figure 3 clearly reveals the evolution of complex asymmetric deformation and strain distribution within the billet during the upsetting–filling stage under the specific configuration of the horizontal extruder, as obtained through finite element simulation. Since the billet was positioned horizontally at the bottom of the extrusion container with an unblocked outlet, its filling process was governed by both gravity and the die geometry, resulting in a highly inhomogeneous distribution of plastic effective strain. Prior to and at Step 69, before the die cavity was completely filled, the effective strain was concentrated in two critical regions. Firstly, the area where the billet contacted and continuously moved upward along the front wall of the die cavity. The high strain in this region originated from severe shear deformation caused by the effects of upward moving under punch pressure. The maximum effective strain in this region reached approximately 1.0, 1.2, and 1.5 at Steps 30, 50, and 69, respectively. Secondly, the area located along an oblique direction connecting the lower right corner of the billet and the bottom of the die’s reduction zone. The strain concentration here resulted from the shear flow of material: while the material at the lower left end was constrained within the dead material zone of the cavity and difficult to move under the punch action, the adjacent material on the right underwent shear flow towards the upper left. These simulation results demonstrate that during the asymmetric upsetting process in this experiment, the material did not undergo simple homogeneous upsetting but experienced significant shear deformation within and between these two critical regions.

**Figure 4:**
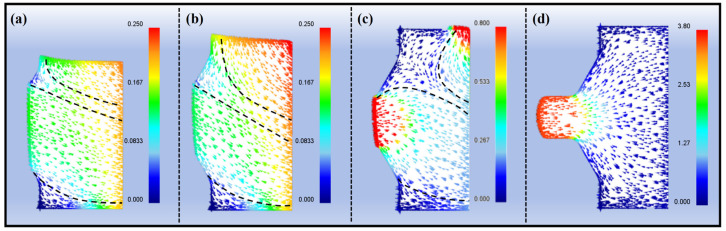
Flow velocity distribution of axial section: (**a**) Step 30, (**b**) Step 50, (**c**) Step 69, (**d**) Step 75.

Figure 4 displays the simulated flow velocity distribution, which clearly reveals the unique deformation characteristics of the asymmetric upsetting process conducted in the horizontal extruder. The simulation indicates that, under the action of the punch, the material exhibits a dominant flow direction from the lower right to the upper left. Due to the the restricted flow of material within the die’s dead zone, and the deceleration of initially free-moving material upon contacting the cavity walls, significant flow velocity differences and shear deformations are induced. This observation corresponds well with the effective strain distribution observed in the high-strain regions in Figure 3. The intense shear deformation intentionally introduced during this filling stage plays a critical and beneficial role in the subsequent specimen. The high-shear-strain zones accumulate substantial dislocations, providing numerous preferential nucleation sites for DRX during the subsequent formal extrusion stage [18]. Additionally, shear deformation helps activate non-basal slip, which can coordinate plastic deformation when basal slip is hindered [19].

Therefore, this asymmetric upsetting process is not merely a simple filling operation but constitutes a crucial pretreatment stage. By proactively utilizing shear deformation, it effectively optimizes the material’s microstructural evolution path, thereby enhancing the overall performance of the final product.

### 3.2. Initial Microstructure

**Figure 5:**
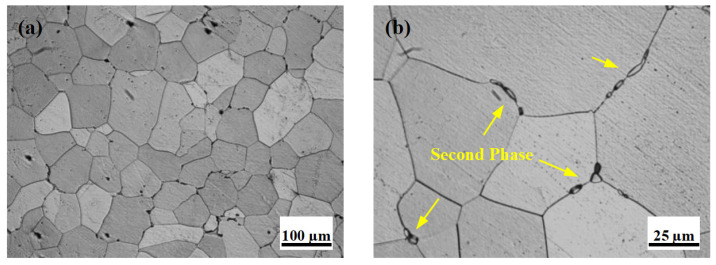
The optical microstructure of the solution-treated sample: (**a**) 100×, (**b**) 400×.

Figure 5 displays the optical microstructures of the alloy following solution treatment at 540 °C for 8 h. The low-magnification panoramic image (Figure 5a) shows that most coarse secondary phase particles dissolved into the α-Mg matrix. A higher-magnification view (Figure 5b) further indicates that a small quantity of residual secondary phases remained undissolved. These phases are primarily distributed discontinuously along the grain boundaries. This microstructural feature suggests that the selected solution treatment parameters were generally appropriate.

**Figure 6:**
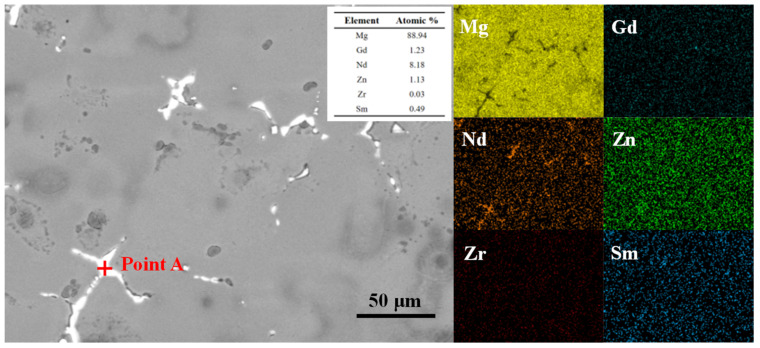
SEM images and EDS analysis results of the solution-treated sample.

To identify the composition of the residual secondary phases along the grain boundaries after solution treatment, the sample was characterized by SEM and EDS. The results are presented in Figure 6. The SEM image clearly reveals the morphology and distribution of the residual phases. EDS elemental mapping indicates a significant enrichment of Nd at the locations of these phases. In contrast, Gd, Zn, and Sm are distributed nearly uniformly across the scanned area. Zr is present in very low amounts and is also uniformly distributed in the matrix. To further determine the chemical composition of these phases, spot EDS analysis was conducted on the largest residual particle in the image. The results show that the atomic percentage ratio of Mg to rare-earth (RE) element is approximately 8.98. This stoichiometric ratio suggests that the residual phase is a Mg-RE intermetallic compound with a composition close to Mg_41_RE_5_ [20].

### 3.3. Extruded Microstructure and Mechanical Properties

**Figure 7:**
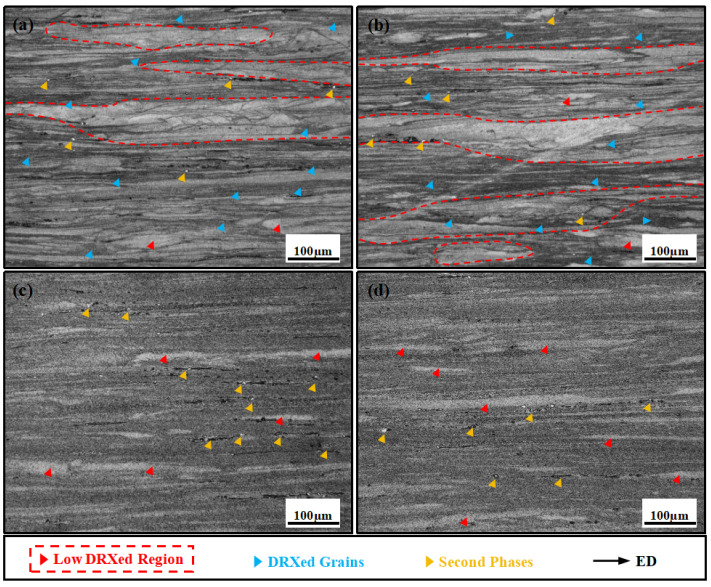
Metallographic structure of the AUEed specimen: (**a**,**b**) 370 °C; (**c**,**d**) 410 °C.

Figure 7a,b shows the optical microstructure of the alloy after AUE at 370 °C. Figure 7a shows numerous coarse, elongated unDRXed grains, along with a large number of extremely fine DRXed grains distributed around them. Although the fine grains are numerous in quantity, the coarse deformed grains still account for the majority in terms of area fraction. As shown in Figure 7b, the grain structure bears a close resemblance to that in Figure 7a. Both are characterized by deformed grains elongated along ED, which alternate with layers of fine-grained regions, collectively exhibiting a bimodal grain structure. In contrast, as displayed in Figure 7c,d, when the deformation temperature was elevated to 410 °C, the microstructure underwent a remarkable transition. The fraction of deformed regions was significantly reduced, accompanied by a substantial improvement in microstructure homogeneity. Although some residual elongated deformed grains persist, the bimodal structure is now predominantly composed of fine DRXed grains, indicating that the increased temperature profoundly promoted the DRX process and grain refinement. During the process, secondary phase particles flow along ED. Under severe deformation, these particles become fragmented and disperse along grain boundary regions. They can serve as potential nucleation sites, promoting localized recrystallization governed by the particle-stimulated nucleation mechanism. This type of bimodal structure, which combines recrystallized fine-grained regions and unrecrystallized coarse-grained regions, generally imparts high strength to the alloy. The fine grains contribute to strengthening through grain boundary mechanisms, while the coarse deformed grains, containing high-density dislocations, provide additional work hardening capacity.

**Figure 8:**
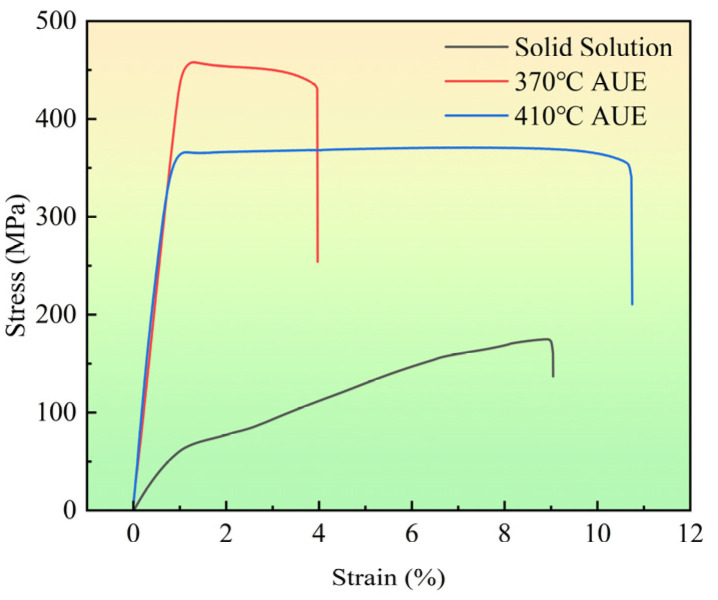
Mechanical properties of solution-treated and AUEed specimens.

The tensile test results in Figure 8 indicate that the alloys after AUE deformation exhibit a significant increase in UTS compared to the solution-treated condition. Specifically, the sample processed at 370 °C achieves an ultra-high UTS of 457.9 MPa, while the sample deformed at 410 °C attains a UTS of 366.1 MPa, both of which are substantially higher than that of the solution-treated sample (174.9 MPa). However, a marked difference in ductility is observed: the EL of the 370 °C deformed sample is considerably lower (3.96%) than that of the solution-treated sample (9.04%), whereas the 410 °C sample exhibits a improved EL of 10.7%. This distinct strength-ductility relationship is intrinsically linked to their characteristic microstructures. The ultra-high strength of the 370 °C sample is primarily attributed to the high-density dislocation strengthening within the coarse deformed grains, which was induced by severe plastic deformation. During the deformation process, dislocations multiply and become entangled, resulting in strong work hardening. This effect is the primary contributor to the enhanced strength. At the same time, the high-density dislocation structure strongly restricts further dislocation motion, leading to a pronounced decline in plastic deformation capability. Moreover, the boundaries of the fine DRXed grains act as strong barriers to dislocation glide. The deformation compatibility between these two distinct microstructural constituents is relatively poor. Stress concentrations tend to develop at their interfaces, facilitating early crack initiation and propagation [21]. In contrast, the more homogeneous microstructure dominated by fine DRXed grains in the 410 °C sample favors better strain accommodation, thereby resulting in better ductility, albeit at the expense of some strength due to the reduced fraction of work-hardened coarse grains. Therefore, although both deformed alloys are significantly strengthened, the 370 °C sample achieves a remarkable strength, the subsequent analysis in this work will focus on elucidating the underlying mechanisms responsible for this ultra-high strength. The remarkable gains in strength make it a promising candidate for structure applications where high specific strength is the primary purpose. Examples include lightweight brackets or supports in aerospace, where weight reduction is critical and the component is primarily subjected to static or steady loads [22].

**Figure 9:**
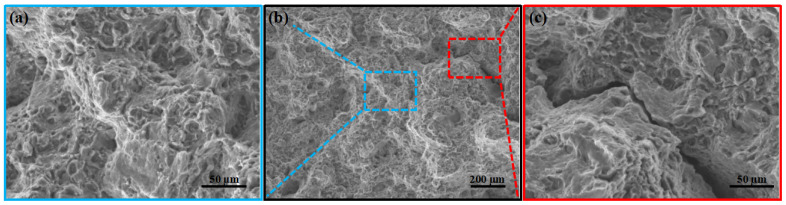
SEM image of the tensile fracture surface of the AUE370 specimen: (**a**) Crack-free microstructure, (**b**) Macroscopic morphology, (**c**) Microstructure with cracks.

To observe the tensile fracture characteristics and deformation mismatch between coarse and fine grains, Figure 9 presents the typical SEM morphology of the fracture surface of the 370 °C AUE (AUE370). Figure 9b shows a low-magnification panoramic view of the fracture surface, which appears uneven with clear tear ridges and localized deformation features. Under higher magnification, Figure 9a, taken from a region without obvious macroscopic cracks, reveals a mixed fracture mode consisting of cleavage facets and dimples. The coexistence of numerous cleavage facets, cleavage steps, and shallow, small dimples suggests limited plastic deformation during fracture. In contrast, Figure 9c clearly displays a large macroscopic crack. The fracture mechanisms on the two sides of this crack exhibit significant asymmetry. The region on the left side consists mainly of flat cleavage facets and steps with very few dimples, indicating a predominantly brittle fracture behavior. On the right side, however, a notably higher density of dimples is observed, reflecting relatively better microplastic deformation capability. Severe dislocation pile-ups in coarse grains lead to stress concentrations that cannot be effectively relaxed by plastic deformation, making these regions prone to cleavage cracking [23]. The fine-grained region promotes uniform plastic deformation, provides nucleation points for ductile tearing, and hinders the propagation of brittle cracks, thus shifting the fracture mechanism from the cleavage path to the dimple path. In summary, this large crack is clearly located between a coarse deformed grain and some fine DRXed grains. Its rapid propagation provided the primary path for final fracture.

### 3.4. Dynamic Recrystallization and Strengthening Mechanisms

**Figure 10:**
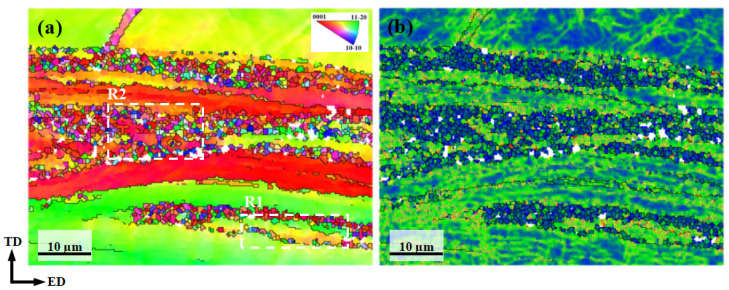
EBSD analysis of AUE370 sample: (**a**) IPF map, (**b**) KAM map.

Figure 10 presents the microstructure and local misorientation analysis of the AUE370 alloy obtained by EBSD. The inverse pole figure (IPF) map in Figure 10a shows a typical heterogeneous deformed microstructure. The unDRXed coarse grains are noticeably elongated along the ED and exhibit clear color gradients inside, indicating high stored energy and the formation of substructures within these grains [24]. These unDRXed grains are surrounded by numerous fine, equiaxed DRXed grains. From the kernel average misorientation (KAM) map in Figure 10b, the interiors of the unDRXed grains show high KAM values, reflecting the accumulation of high-density dislocation tangles and numerous low-angle grain boundaries. In contrast, the fine DRXed grains exhibit very low KAM values, demonstrating their recrystallized nature with low dislocation density and minimal internal stress. The highest KAM values are localized near high-angle grain boundaries and subgrain boundaries inside the deformed grains. This indicates that dislocation accumulation occurs near these interfaces, resulting in high local lattice distortion and strain energy storage [25,26]. In summary, the pronounced microstructure confirms that DRX was not fully achieved during the deformation process at 370 °C. The fine DRXed grains contribute to strength via boundary strengthening mechanisms. Meanwhile, the coarse deformed grains, which contain high-density dislocations and substructures, provide strong work hardening capacity. The synergy between these two distinct regions leads to the ultra-high strength of the alloy.

**Figure 11:**
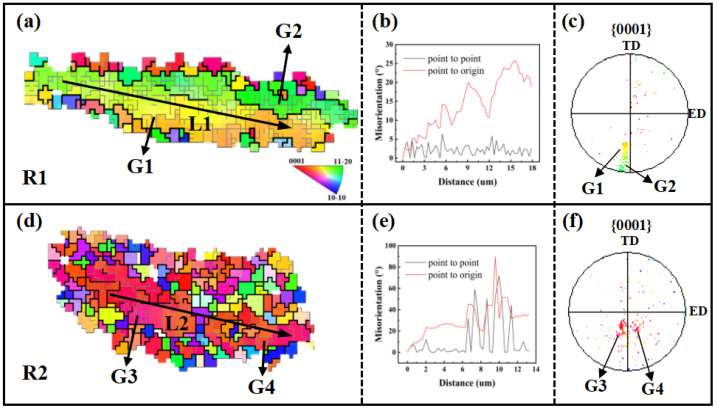
The typical regions selected in Figure 10a: (**a**,**d**) IPF map, (**b**,**e**) line graph of misorientation angle along the L1 and L2, (**c**,**f**) represent {0001} pole figure of regions R1 and R2.

To further investigate the DRX mechanisms in the AUE370 alloy, two typical regions (R1 and R2) indicated by the white dashed boxes in Figure 10a were selected for detailed EBSD analysis, with the results presented in Figure 11. In the elongated grain zone, designated as region R1 and illustrated in Figure 11a–c, a continuous gradient in inverse pole figure coloring is evident within the deformed grain G1. Analysis of local misorientation along the path indicated by arrow L1 in Figure 11b shows that point-to-point misorientations remain below 10°, whereas the cumulative misorientation surpasses 20° [27,28]. Furthermore, grain G2 displays a crystal orientation highly similar to that of G1, as seen in Figure 11c Together, these three observations strongly support the conclusion that continuous dynamic recrystallization (CDRX), is the dominant mechanism in this region, characterized by the gradual rotation of subgrains to form new grains [29,30]. Moving to the fine-grained region R2, presented in Figure 11d–f, partially recrystallized grains G3 and G4 still exhibit clear orientation gradients, shown in Figure 11e. In addition, most of the surrounding DRXed grains share similar orientations with the two grains, as demonstrated in Figure 11f, which further corroborates the central role of CDRX during the recrystallization process. In summary, grain refinement in the present deformation process is mainly attributable to the CDRX mechanism, which enables a continuous transition from the deformed matrix to a recrystallized microstructure through intragranular dislocation rearrangement and the evolution of subgrain boundaries.

**Figure 12:**
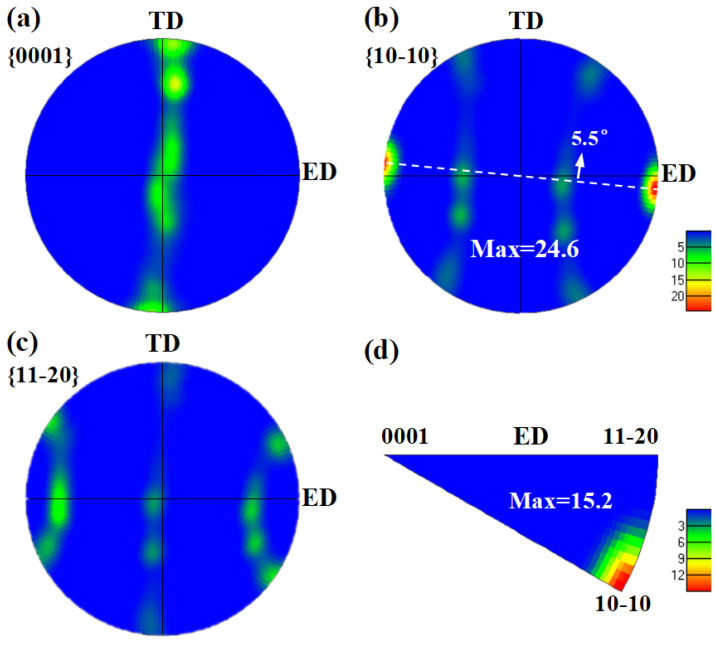
AUE370 sample: (**a**–**c**) pole figures; (**d**) inverse pole figure.

Figure 12 systematically characterizes the macro-texture of the AUE370 alloy through (a) {0001}, (b) {10-10}, and (c) {11-20} pole figures, along with (d) an IPF map parallel to the ED. The analysis results indicate that the sample developed a strong <10-10>//ED fiber texture, which is a common basal fiber texture observed in magnesium alloy extrusion [13,14,31,32,33]. Analysis of Figure 12a–c indicates that the fiber texture in the present alloy is rotated approximately 5.5° relative to that typical fiber texture. This may be because the shear deformation introduced in the upsetting deformation stage changes the distribution of the texture. Simultaneously, in the Figure 12d, the maximum pole density is precisely located at the <10-10> position. Together, these findings confirm that the <10-10> direction of the grains is nearly parallel to the ED. The core characteristic of this texture is that the c-axes of the vast majority of grains are radially aligned and perpendicular to the ED, thereby causing the basal planes to be approximately parallel to ED. This strong basal fiber texture has a decisive influence on the mechanical behavior of the alloy. During tensile testing along the ED, the basal planes of most grains are parallel to the stress axis. This results in a Schmid factor (SF) for basal slip that is pretty low, making it difficult to activate [34,35]. Consequently, this texture significantly enhances the YS and UTS.

**Figure 13:**
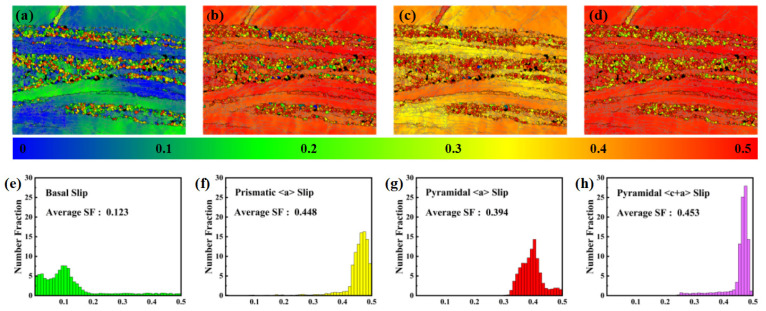
Schmid factor distribution maps and Schmid factor distribution histograms when an external force is applied along the ED: (**a**,**d**) Basal slip, (**b**,**e**) Prismatic <a> slip, (**c**,**f**) Pyramidal <a> slip; (**d**,**h**) Pyramidal <c+a> slip.

To further investigate the influence of the strong <10-10>//ED texture on the tensile deformation mechanisms of the AUE370 alloy along the ED, the SF distributions for the major slip systems under tensile loading were calculated, with the results shown in Figure 13. The SF maps and histograms clearly reveal the decisive role of texture on the activation propensity of the slip systems. For basal slip (0001)<11-20> (Figure 13a,d), the SF distribution is dominated by low values (blue and green), with an average SF of merely 0.123. This indicates that the basal planes of the vast majority of grains are indeed parallel to the tensile axis (ED), resulting in an extremely low resolved shear stress component for basal slip. Consequently, basal slip is strongly suppressed during the tensile deformation. In contrast, the average SF values for prismatic <a> slip, pyramidal <a> slip, and pyramidal <c+a> slip are significantly higher, at 0.448, 0.394, and 0.453, respectively. This indicates that these non-basal slip systems become the primary carriers of plastic deformation. This unique SF distribution originates from the strong basal fiber texture, which forces plastic deformation to transition from the typically easiest-to-activate basal slip to non-basal slip systems (particularly prismatic <a> and pyramidal <c+a> slip) that require higher CRSS [36,37]. This shift in deformation mechanism is another major reason for the ultra-high YS and tensile strength observed along the ED. However, the limited slip capacity and higher activation stresses of these non-basal slip systems severely constrain plastic deformation [38]. They also readily induce stress concentrations, ultimately leading to a significant reduction in macroscopic ductility. This analysis provides an explanation for the high-strength, low-ductility mechanical behavior observed in Figure 8, forming a complete chain of evidence alongside the observed texture characteristics.

## 4. Conclusions

(1) Finite element simulation reveals a unique shear deformation mode during the asymmetric upsetting–filling stage. Under the combined effects of gravity and die constraints, the billet undergoes intense asymmetric shear strain and flow, which introduces high deformation stored energy in advance. This lays the foundation for subsequent DRX nucleation and texture evolution during subsequent deformation.
(2) Utilizing a process combining low-speed extrusion with a high extrusion ratio at 370 °C and asymmetric upsetting, an ultra-high strength magnesium alloy with an ultrafine-grained bimodal microstructure was successfully fabricated at 370 °C. This constitutes one of the primary reasons for its high UTS of 457.9 MPa.
(3) CDRX is the primary grain refinement mechanism during AUE370. It significantly refined a portion of the grains. However, it failed to completely eliminate the coarse deformed grains, resulting in the formation of a bimodal microstructure.
(4) The strong <10-10>//ED basal fiber texture is one of the key factors responsible for the high strength and low ductility of the AUE370 alloy. This texture strongly inhibits the activation of basal slip (average SF merely 0.123) and forces plastic deformation to rely on harder-to-activate non-basal slip systems.

## Data Availability

The original contributions presented in this study are included in the article. Further inquiries can be directed to the corresponding authors.

## Abbreviations

The following abbreviations are used in this manuscript:

UE	Upsetting extrusion
UTS	Ultimate tensile strength
EL	Elongation
DRX	Dynamic recrystallization
YS	Yield strength
ED	Extrusion direction
CRSS	Critical resolved shear stresses
AUE	Asymmetric upsetting–extrusion
OM	Optical microscopy
SEM	Scanning electron microscopy
EBSD	Electron backscatter diffraction
RE	Rare-earth
AUE370	370 °C AUE
IPF	Inverse pole figure
KAM	Kernel average misorientation
CDRX	Continuous dynamic recrystallization
SF	Schmid factor
